# Transdiagnostic Sleep and Circadian Intervention in Youth: Long-Term Follow-Up of a Randomized Controlled Trial

**DOI:** 10.1016/j.jaac.2024.05.001

**Published:** 2024-05-08

**Authors:** Eli S. Susman, Estephania Ovalle Patino, Sondra S. Tiab, Lu Dong, Nicole B. Gumport, Laurel D. Sarfan, Stephen P. Hinshaw, Allison G. Harvey

**Affiliations:** University of California, Berkeley, California; University of California, Berkeley, California; University of California, Berkeley, California; RAND, Santa Monica, California; Stanford University, Stanford, California; University of California, Berkeley, California; University of California, Berkeley, California; University of California San Francisco, San Francisco, California; University of California, Berkeley, California

**Keywords:** eveningness, habit formation, psychopathology, sleep, young adults

## Abstract

**Objective:**

This preregistered study compared the effects of the Transdiagnostic Sleep and Circadian Intervention (TranS-C) with psychoeducation (PE) about sleep, health, yoga, meditation, and outdoor appreciation activities on sleep and circadian functioning, health risk, and sleep health behaviors at long-term follow-up (LTFU), an average of 8 years following treatment. We also examined if more sleep health behaviors at LTFU were associated with better sleep and circadian functioning at LTFU and if better sleep and circadian functioning were associated with lower health risk at LTFU.

**Method:**

At baseline, we randomly assigned adolescents with an eveningness chronotype to TranS-C (n = 89) or PE (n = 87). Of this sample, we assessed 106 young adults (mean age at follow-up = 22.5 years; n = 55 from TranS-C; n = 51 from PE) an average of 8 years following treatment.

**Results:**

Despite TranS-C (vs PE) sustaining improvement in circadian functioning through 12-month follow-up, at LTFU, there were no significant differences between the conditions on any outcome, including sleep and circadian functioning, risks in 5 health domains indexed by self-report and ecological momentary assessment, sleep health behaviors, and physical measurements. Across both conditions, measures indicating poorer sleep and circadian functioning were associated with higher health risk across multiple domains, and more sleep health behaviors were associated with lower levels of eveningness at LTFU.

**Conclusion:**

These results provide an important window into the influence of development on long-term outcomes for youth and raise the possibility that interventions for youth could be enhanced with a focus on habit formation.

**Clinical trial registration information:**

Maintaining Behavior Change: A 6-Year Follow-up of Adolescent ‘Night-owls’; https://www.clinicaltrials.gov/; NCT05098782.

## Plain language summary

This study evaluated the effects of a psychological treatment (the Transdiagnostic Sleep and Circadian Intervention [TranS-C]), relative to a psychoeducation active control (PE) at 8 years following treatment, on sleep problems, the preference and tendency for later bedtimes and health risk (ie, risk for worse health). Across both groups combined, the study also examined whether more sleep health behaviors are related to better sleep and whether better sleep is related to lower health risk. At long-term follow-up, there were no differences between TranS-C and PE on any outcome. However, overall poorer sleep was related to greater health risk, and those who engaged in better sleep habits tended to have an earlier “feeling best” sleep rhythm at long-term follow-up. Given evidence that youth tend to outgrow their preference and tendency for later bedtimes with age, our findings shed light on the potential impact of development on long-term outcomes for youth and raise the question of whether treatments for youth could be improved with evidence-based tools for promoting habit formation of positive sleep behaviors.

An implicit goal of evidence-based psychological treatments (EBPTs) is to reverse the processes that maintain psychological problems by fostering health-promoting behavior.^1^ Exciting advancements have been made in EBPTs, which are considered first-line interventions for many conditions.^2^ Yet, approximately half of EBPT recipients do not recover.^3^ One-third do not demonstrate meaningful benefits.^3^ Even for recipients who benefit, a drop in gains over the years after treatment is common.^4^

We note 5 distinct trajectories following the receipt of intervention: a fadeout of treatment effects, where improvement after intervention is not maintained at follow-up (eg, ^4^); an upward spiral effect, where improvement after intervention becomes more pronounced at follow-up (though effects may taper off at longer-term follow-up) (eg, ^5^); a logarithmic effect, where gains are achieved after intervention and maintained at follow-up (eg, ^6^); null effects after intervention with positive outcomes only at follow-up (eg, ^7^); or null effects both after intervention and at follow-up (eg, ^3^).

For interventions delivered in youth, the failure to sustain gains through young adulthood is particularly common.^8^ Indeed, several studies have documented postintervention gains in youth followed by a fadeout over time.^4,9,10^ One explanation could be that interventions that lead to short-term changes rarely form habits that are sustained over the long term.^1,11^ Another possibility is that fadeouts are influenced by development. A fadeout relative to a control condition may be observed if the intervention target without intervention changes with development. To state this another way, although sometimes helpful in the short term, interventions that target a process that would have progressed with age may not show long-term differences relative to a control group. This trajectory has been documented for tics, problematic alcohol use, and mild to moderate oppositional defiance, as youth may outgrow these with age.^9,10,12^ We aimed to evaluate a long-term follow-up (LTFU) of young adults who received a psychological intervention to modify eveningness chronotype in adolescence.

Eveningness chronotype is typically defined as a preference for one’s activity (feeling best rhythm) and sleep entrainment to occur at later hours.^13^ In adolescents and young adults, eveningness is common (eg, ^14^), has been associated with more sleep problems cross-sectionally^15^ and longitudinally (eg, ^16^), and predicts and predates heightened risk for health problems in multiple domains (eg, ^17^). In the emotional domain, eveningness predicts emotional problems (eg, ^18^). In the cognitive domain, it is associated with worse academic performance (eg, ^19^). In the behavioral domain, it predicts poorer self-regulation and greater substance use (eg, ^20^). In the physical domain, it predicts less physical health and exercise (eg, ^21^). In the social domain, it predicts fewer and less positive social ties (eg, ^22^). We refer to these risks (emotional, cognitive, behavioral, physical, and social) as the 5 health-relevant domains.

In the original randomized controlled trial (RCT), we tested whether it is possible to decrease eveningness and improve health outcomes among youth (10–18 years old) at risk in at least 1 of 5 health domains using the Transdiagnostic Sleep and Circadian Intervention (TranS-C).^23^ TranS-C (n = 89 participants) aims to reverse modifiable cognitive and behavioral contributors to eveningness (eg, irregular routines, nighttime technology use).^24^ The comparison group received psychoeducation (PE) (n = 87 participants). In the original RCT, TranS-C, relative to PE, predicted improvement for selected sleep, circadian, and health outcomes at posttreatment, including reduced eveningness.^23^ At 6-month follow-up (6FU) and 12-month follow-up (12FU), relative to PE, TranS-C predicted reduced eveningness and weeknight/weekend discrepancy in sleep diary wake-up time.^25,26^ Pittsburgh Sleep Quality Index (PSQI) scores improved from pretreatment to 6FU.^26^ These findings add to the growing evidence that poor sleep among youth can be improved with psychosocial interventions^27,28^ and that the psychosocial contributors to eveningness are modifiable.^29,30^ Still, although LTFUs of EBPTs for other sleep problems (ie, cognitive-behavioral therapy for insomnia [CBT-I]) have shown success in middle-aged adults,^6^ it is unknown whether an intervention to modify eveningness chronotype in adolescents leads to gains that persist through young adulthood.

We report preregistered analyses for 3 aims. Aim 1 was to assess participants in the initial RCT^23^ for sleep and circadian functioning and functioning in the 5 health-relevant domains. Hypothesis 1a is that youth receiving TranS-C, relative to youth receiving PE, will exhibit better sleep and circadian functioning and lower health risk at LTFU. Hypothesis 1b is that better sleep and circadian functioning at LTFU will be associated with lower health risk at LTFU regardless of treatment arm. Aim 2 was to assess the sample for their engagement in sleep health behaviors (behaviors that promote better sleep health, such as consistent bed and wake times, avoiding naps in the late afternoon and evening, etc). Hypothesis 2a is that youth receiving TranS-C, relative to PE, will utilize more sleep health behaviors and develop stronger sleep health habits at LTFU. Hypothesis 2b is that better sleep and circadian outcomes at LTFU will be associated with more utilization and stronger habits at LTFU regardless of treatment arm. Our exploratory aim on whether treatment effects are moderated by age, sex, and family income is reported in Supplement 1 and Table S1, available online, because the findings are likely underpowered.

## METHOD

### Participants and Procedures

Youth who participated in a previous RCT^23,25,26^ when they were 10 to 18 years old were invited to participate in LTFU. The original RCT recruited participants through clinician referrals and advertisements from January 2013 to February 2016. Participants and caregivers were screened by phone for eligibility. Potentially eligible individuals completed an in-person assessment to confirm their eligibility. Inclusion criteria for the original RCT are provided in Supplement 1 and Table S2, available online. RCT participants and their caregivers provided informed assent and consent. At baseline, we randomly assigned eligible youth with eveningness chronotype (58% female; mean age = 14.8 years) to TranS-C (n = 89) or PE active control (n = 87) using a computerized random number generator, stratified by age and biological sex assigned at birth. We used a parallel RCT design, following Consolidated Standards of Reporting Trials (CONSORT) requirements. Only participants, therapists, and coordinators managing randomization knew of participants’ allocations (TranS-C or PE). RCT procedures are further described elsewhere^23,25^ and registered on ClinicalTrials.gov (NCT01828320).

**FIGURE 1:**
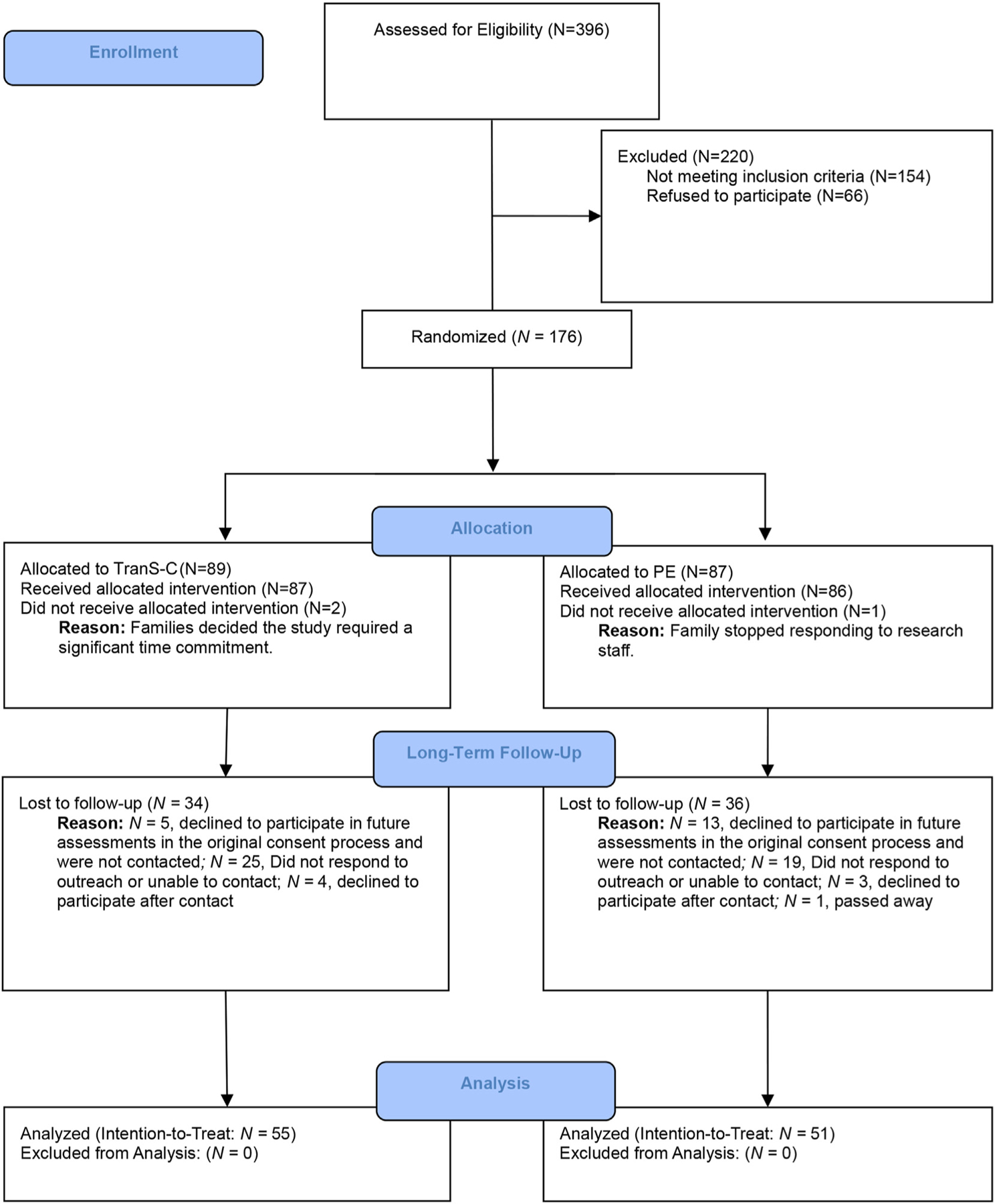
CONSORT Diagram Illustrating the Flow of Participants Through the Study **Note:** PE = psychoeducation; TranS-C = Transdiagnostic Sleep and Circadian Intervention.

Of original RCT participants, 18 declined to participate in future research. LTFU inclusion criteria were individuals who participated in the original RCT. There were no LTFU exclusion criteria. Hence, 158 young adults were invited to participate in LTFU. We recontacted participants from December 2021 to October 2022. Now all ≥18 years old, they provided informed consent. Participants completed an assessment on Zoom (https://zoom.us/) with a trained interviewer, ecological momentary assessment (EMA), a sleep diary, and actigraphy for 7 days. LTFU included 106 participants (58% female; mean age at follow-up = 22.5 years; n = 55 from TranS-C group; n = 51 from PE group) assessed about 8 years after intervention (Table 1). The study is reported following CONSORT requirements (see Figure 1 for participant flow diagram). The Committee for Protection of Human Subjects at the University of California, Berkeley, approved all study protocols. The analyses were preregistered (osf.io/d5a4g). Data and analysis code are publicly available (osf.io/p5jbk). We registered the LTFU protocol at ClinicalTrials.gov (NCT05098782). Although the protocol specified a 6-year follow-up, due to funding, COVID-19-related delays, and longer than anticipated recruitment, the follow-up was about 8 years after the RCT. We believe this change continues to align with the study’s goal—to conduct an LTFU of these youth.

### Treatment Conditions

Both treatments in the original RCT consisted of 6 sessions lasting 50 minutes that were delivered over 6 weeks during the academic year by masters- or doctoral-level therapists. One important difference between TranS-C and PE is that TranS-C is designed to facilitate behavior change, whereas PE provides information without scaffolding behavior change. This differentiation is supported by qualitative coding of therapist actions.^23^ More information about the conditions is provided briefly below and in more detail elsewhere.^23,25^

#### Transdiagnostic Sleep and Circadian Intervention for Youth.

TranS-C addresses a range of sleep and circadian problems across various psychosocial, behavioral, and cognitive processes in youth.^23^ TranS-C is composed of 4 core modules (behavioral components, daytime impairment, unhelpful beliefs, relapse prevention), 4 cross-cutting modules (functional analysis, goal setting, motivational interviewing, education), and 7 optional modules (bedtime worry, sleep efficiency, time in bed, delayed/advanced phase, continuous positive airway pressure compliance, environment, nightmares).

#### Psychoeducation.

PE is an active control.^31^ PE includes information on sleep, health, accidents, stress, diet, exercise, and mood. Participants are invited to practice yoga, meditation, and outdoor appreciation activities. The emphasis is on alliance building, reflective listening, and education without emphasizing behavior change.

#### Text Message Intervention.

At 6FU, we randomly assigned participants in both TranS-C and PE to receive text messages that repeated treatment information (n = 47), text messages that prompted recall of treatment information (n = 50), or no text messages (n = 47). The content of the repeated treatment information and prompted recall of treatment information text messages differed by treatment condition (TranS-C vs PE) and were tailored to the TranS-C vs PE protocol. This intervention was implemented and examined between 6FU and 12FU.^25^ Our primary interest was testing TranS-C vs PE differences at LTFU. We did not evaluate text message effects at LTFU.

### Measures

#### Sleep and Circadian Outcomes.

We used several measures to assess sleep and circadian functioning at LTFU. The Composite Scale of Morningness (CSM) is a primary outcome of LTFU (13 items, 4- or 5-point scales).^32^ Lower scores indicate greater eveningness. Eveningness chronotype is composed of 2 parts: later circadian timing and a preference to be awake at later hours.^13^ Although CSM correlates with actigraphy sleep midpoint, we opted for a self-report measure to capture both components of eveningness.^32^ The Patient-Reported Outcomes Measurement Information System–Sleep-Related Impairment (PROMIS-SRI) is another primary outcome assessing sleep-related impairment using (8 items, 5-point scale).^33^ The PROMIS–Sleep Disturbance (PROMIS-SD) is a primary outcome assessing sleep disturbance (8 items, 5-point scale).^33^ These measures have demonstrated excellent reliability and validity.^32,33^ For all PROMIS measures, we used T scores, calculated from the sum of raw scores using scoring manuals.^34^

The PSQI^33^ is a secondary outcome assessing sleep quality over the past 4 weeks (19 items, 4-point scale and integer responses). The midpoint fluctuation of sleep is a secondary outcome indexing the temporal stability of chronotype (sleep entrainment), which we assessed using actigraphy (Actiwatch GT9X Link; Philips Respironics). The rationale for selecting this outcome is twofold. First, high actigraphy midpoint fluctuation is positively associated with adverse outcomes.^35^ Second, as circadian functioning is a key goal of TranS-C, we included this objective, naturalistic index to complement self-report. Actigraphy assesses movement samples in 60-second epochs and is validated in youth.^36^ We calculated the intraindividual variability using the estimated within-subject standard deviation.^37^ The Sleep Health Composite score is a secondary outcome defined as the sum score of 6 sleep health dimensions: regularity (midpoint fluctuation), satisfaction (sleep quality question on PROMIS-SD), alertness (daytime sleepiness question on PROMIS-SRI), timing (mean midpoint), efficiency (sleep diary sleep efficiency), and duration (sleep diary total sleep time).^38^ Higher scores indicate better sleep health. Information on the derivation of the Sleep Health Composite is provided in Supplement 1, available online.

#### Five Health-Relevant Domains.

We calculated 2 clusters of composite scores using the cumulative risk index for each of the 5 health-relevant domains, composed of emotional, cognitive, behavioral, social, and physical health.^39^ The first cluster is the self-report composite risk score, which comprises primary outcomes calculated from a combination of validated questionnaires assessing each of the 5 health-relevant domains. The second cluster is the EMA composite risk score. These are secondary outcomes collected over 7 days via text messages 2 times a day on weekdays and 4 times a day on weekends to index real-world functioning in the health-relevant domains (eg, for the cognitive domain, concentration, distractedness, and focus are rated). The questions are adapted from prior research.^23^ More information on the derivation of the self-report and EMA composite risk scores is in Supplement 1, available online.

#### Sleep Health Behaviors.

The utilization scale^11^ is a primary outcome assessing utilization of sleep health behaviors at LTFU based on how often 14 sleep health behaviors were performed in the last week (0–4 scale). The Self-Report Behavioral Automaticity Index (SRBAI)^40^ integrated with the utilization scale (SRBAI-US) is a primary outcome used to assess habit formation of sleep health behaviors at LTFU (5-point scale). The SRBAI-US uses a stem (“Behavior X is something …”), measuring sleep and circadian functioning followed by 1 item assessing habit formation (“… I do automatically”). “Behavior X” is substituted with the 14 sleep health behaviors assessed (eg, I try to wake up about the same time each morning).

#### Physical Measurements.

Body mass index (calculated as weight in kilograms divided by height in meters squared) and waist circumference are other outcomes. We measured waist circumference midway between the lowest rib and the superior border of the iliac crest with a flexible tape. If the first 2 measurements differed by >1 cm, we took 2 more measurements. We used the average of all waist circumferences.

### Data Analysis

We conducted analyses in R version 4.1.2 (https://www.R-project.org/). For all analyses, we covaried participants’ sex assigned at birth, age at LTFU, and text messaging intervention (dummy-coded as 0 = none, 1 = repeated treatment information, 2 = prompted recall of treatment information).^25^ Although we recontacted all participants who completed the original RCT and consented to be contacted for future research, regardless of how much treatment they received, we analyzed only the participants who completed the long-term follow-up, so the analyses used a modified intention-to-treat approach.^41^ Nonbinary variables were modeled as continuous.

For aims 1a and 2a, we used multiple linear regression with sleep and circadian functioning and health risk as the outcome variables. We assigned treatment (TranS-C vs PE) as the independent variable. For EMA, we used multilevel modeling to contrast TranS-C with PE at LTFU and account for multiple observations nested within participants. Outcomes were modeled as continuous except for EMA physical and EMA social health risk, coded as binary (1 = was physically active today or socializing right now, respectively, 0 = was not active/socializing). Multilevel logistic regression was used for binary outcomes. The fixed component of all multilevel models included the above-mentioned covariates and indicator variables for treatment (TranS-C = 1, PE = 0). The random part of all multilevel models included a subject-specific random intercept and a subject- and occasion-specific error term.

For aims 1b and 2b, we used multiple linear regression for self-reported health risk and multilevel modeling for EMA, as described above, with the sleep and circadian measures serving as the independent variable and assigned treatment (TranS-C = 1, PE = 0) serving as a covariate. See Supplement 1, available online, for information on exploratory analyses.

#### Power Consideration.

Because we were constrained by the number of original RCT participants who agreed to take part in LTFU, we calculated that at least a medium effect size (*d* ≥ 0.55) for a continuous outcome could be detected with our response rate (61%, 106 out of 176 original RCT participants), 80% power, and 2-tailed α of .05 using G*Power version 3.1.9.6 (https://www.psychologie.hhu.de/arbeitsgruppen/allgemeine-psychologie-und-arbeitspsychologie/gpower.html). If the minimum detectable effect size = 0.50, power = 72%; if 0.40, power = 53%; if 0.35, power = 43%; if 0.30, power = 33%.

Given that the response rate was lower than expected (from the planning stage of this study), we consider the analyses of TranS-C vs PE outcomes at LTFU exploratory in nature; null results could be due to insufficient power to detect small/small-to-medium range effect sizes (*d* < 0.55). Given the exploratory nature, we did not correct for multiple testing, and we acknowledge this can lead to an inflated type I error rate. We emphasize interpreting the degree of certainty of the estimates by reporting standardized effect size and 95% CI^42^ in all analyses (Tables 2–4; Table S1, available online). We also encourage considering effect sizes alongside their associated power (see preceding paragraph). Further information on how effect sizes were calculated is in Supplement 1, available online. We report absolute effect sizes of binary outcomes in Table S3, available online.

## RESULTS

### Demographic and Descriptive Statistics

Table 1 presents demographic and participant characteristics. Table S4, available online, presents descriptive statistics for all outcome variables. Participants who dropped out of LTFU were not significantly different from participants who completed LTFU on sex assigned at birth (𝜒^2^_1_= 0.04, *p =* .838), pretreatment family income (𝜒^2^_9_ = 12.00, *p* = .213), text condition (𝜒^2^_4_ = 6.00, *p =* .199), or treatment condition (𝜒^2^_1_ = 0.95, *p =* .330). However, participants who completed LTFU were 6.77 months younger at pre-treatment than dropouts (*t*_142.80_ = 1.99, *p =* .049). Table 1 presents TranS-C vs PE differences regarding participant characteristics at LTFU. Other than more participants identifying as Asian in TranS-C relative to PE at LTFU, there were no significant differences between the 2 conditions (Table 1). The number of participants identifying as Asian from the original RCT who did not participate in LTFU was not significantly different between the 2 conditions (𝜒^2^_1_ = 2.86, *p* = .091). Moderation analyses of treatment effects are reported in ,Table S1 available online, and are generally null.

### Sleep and Circadian Outcomes and the 5 Health-Relevant Domains (Aim 1a)

Sleep and circadian outcome data are provided in Table 2. There were 3 primary sleep and circadian outcomes: CSM, PROMIS-SRI, and PROMIS-SD. There were 3 secondary sleep and circadian outcomes: PSQI, midpoint fluctuation of sleep, and composite sleep health score. There were no significant differences at LTFU between TranS-C and PE on these outcomes. These models identified an association of age (a covariate) with CSM that was not observed with any other sleep or circadian outcome, such that older LTFU participants exhibited higher CSM scores (less eveningness/more morningness) (*β* = .22, 95% CI [0.03, 0.42], *p =* .027).

Table 2 shows TranS-C vs PE effects on the 5 health-relevant domains at LTFU. There were 5 primary health outcomes—self-report composite risk scores in the 5 health domains. There were 5 secondary health outcomes—EMA composite risk scores in the 5 health domains. We examined BMI and waist circumference as other outcomes. There were no significant TranS-C vs PE differences in these outcomes at LTFU.

### Associations of Sleep and Circadian Functioning With Health Risk (Aim 1b)

Table 3 provides results on the associations of sleep and circadian functioning with health risk at LTFU across treatment conditions (TranS-C and PE). We tested each sleep and circadian outcome as a predictor of self-report and EMA composite risk scores in the 5 health domains. First, self-report emotional health risk was associated with worse CSM, PROMIS-SRI, PROMIS-SD, PSQI, and Sleep Health Composite scores. Second, self-report cognitive health risk was associated with worse CSM, PROMIS-SRI, PROMIS-SD, PSQI, and midpoint fluctuation of sleep. Third, self-report behavioral health risk was associated with worse CSM, PROMIS-SRI, PROMIS-SD, PSQI, and midpoint fluctuation of sleep. Fourth, self-report social health risk was associated with worse PROMIS-SD and PSQI. Fifth, self-report physical health risk was associated with worse PROMIS-SD and PSQI.

EMA emotional health risk was associated with worse PROMIS-SRI, PROMIS-SD, and PSQI. EMA cognitive health risk was associated with worse PROMIS-SRI. EMA behavioral health risk was associated with worse PSQI and sleep health composite. EMA social and physical health risk were not significantly associated with sleep and circadian outcomes.

### Sleep Health Behaviors Outcomes (Aim 2a)

Sleep health behavior outcome data are provided in Table 2. There were 2 primary sleep health behavior outcomes: utilization and habit formation of sleep health behaviors. There were no significant differences between TranS-C and PE on these outcomes.

### Sleep and Circadian Functioning Associated With Sleep Health Behaviors (Aim 2b)

Results on the associations between utilization and habit formation of sleep health behaviors with sleep and circadian functioning at LTFU are provided in Table 4. Greater utilization and habit formation of sleep health behaviors were associated with higher levels of morningness, as indexed by CSM. We found no other significant associations.

## DISCUSSION

In prior research, TranS-C (vs PE) sustained improvements in circadian functioning through 12FU.^23,25,26^ This study examined the effects of TranS-C vs PE on sleep and circadian functioning, health risk, and sleep health behaviors at approximately 8 years after intervention (LTFU). We also sought to determine whether more sleep health behaviors at LTFU were associated with better sleep and circadian functioning at LTFU and whether better sleep and circadian functioning at LTFU were associated with lower health risk. Interestingly, there were no significant TranS-C vs PE differences at LTFU (aim 1a). This is a concerning negative result because a pattern of poorer circadian functioning (CSM, midpoint fluctuation, and/or Sleep Health Composite) was associated with higher emotional, cognitive, and behavioral health risk, and a pattern of poorer sleep functioning (PROMIS-SRI, PROMIS-SD, PSQI, and/or Sleep Health Composite) was associated with higher risk across all health domains (including social and physical; aim 1b).

Several possibilities might explain the observed fade-out in treatment effects. One developmental contributor could be that youth from both conditions outgrew their eveningness with age. Although a 10-year follow-up of CBT-I showed lasting benefits,^6^ it was delivered during a developmental period (middle adulthood) in which the treatment target was unlikely to change without intervention. Preference for eveningness peaks around late adolescence (ages 16–20) before shifting back toward morningness in young adulthood.^43,44^ Indeed, in our sample (now 18–27 years old), levels of eveningness were lower in older participants than younger participants. Also, although 100% of RCT youth were in the lowest quartile of the Children’s Morningness-Eveningness Preference Scale (CMEP) at pretreatment (compared with 25% of similar-aged peers exhibiting this level of eveningness in the general population),^23,45^ only 35% to 53% of those in either treatment group scored in the lowest quartile of our adult measure of eveningness (the CSM^32^) by LTFU (compared with 25% of similar-aged peers) (Table 1). The suggestion is that both treatment groups showed improvement that could be related to the passage of time or development. However, determining the specific temporal or developmental factors (eg, changes in puberty, lifestyle, work, education, health/ medical conditions, significant life events) that could have contributed to such improvement in both conditions remains a fascinating question for future research. The findings extend the literature on youth psychological treatments that show a fade-out in long-term effects when the treatment target would have resolved without intervention.^9,10,12^

Does this mean it is not worth treating such issues in adolescence? We propose that even if the effects of TranS-C vs PE (eg, reductions in eveningness and weeknight/weekend discrepancy for wake-up time, as demonstrated in prior research)^11,25^ last only through 12FU, that alone may still be meaningful. However, it remains an empirical question if that somewhat transient behavior change in responders pays dividends in the future. For example, TranS-C, or its associated improvements in circadian functioning at 12FU,^25^ could have led to improved health or other outcomes at an interval shorter than 8 years (eg, 4 years post-intervention). Future studies detailing the trajectory of behavior change after intervention are needed to better understand how and when outcomes begin to decline. This could inform methods to support long-term change (eg, booster sessions).

Another possible contributor to the fade-out is that PE could have continued to exert positive effects, as it did through 12FU.^23,25,26^ PE provides sleep education and the opportunity to practice yoga, meditation, or outdoor appreciation activities, which can confer real benefits.^23,25,26,31^ As a stand-alone intervention, the effects of mindfulness on sleep have demonstrated noninferiority to CBT-I and persist for at least several years, yet take more time than CBT-I to fully manifest.^46,47^ Although unlikely to have been the only factor, this raises the possibility that a sustained or upward spiral effect from PE could have been one of the contributors to the observed fade-out of TranS-C.

TranS-C did not foster more sleep health behaviors (utilization and habit formation) than PE at LTFU (aim 2a). Yet, more sleep health behaviors were associated with less eveningness across both conditions at LTFU (aim 2b). Research is needed on how to sustain more sleep health behaviors, as their negative association with eveningness adds to the growing literature that more healthy behavior is associated with better outcomes (eg,^48^). However, the finding that more sleep health behaviors was associated with less eveningness is somewhat discrepant with prior time points. At 12FU, higher utilization was not associated with self-reported eveningness (ie, later circadian timing and preference on the CMEP), although it was associated with earlier sleep diary bedtime (ie, earlier timing, in practice).^11^ Youth could become more likely to maintain sleep health behaviors that foster an earlier feeling best rhythm as self-regulation and values-based decision making develop with age.^49^ This could be one of many developmental factors contributing to the suggested improvement in both treatment groups and the negative association between eveningness and age.

There are some limitations. First, although this study extends the literature by evaluating a transdiagnostic modular treatment relative to an active control at about 8 years after treatment, longer follow-ups tend to incur more attrition.^50^ This level of attrition (39%) limited our power, which likely ranged from 33% to 86% to detect potential small effect-size differences (*d* > 0.30) and could have reflected response biases. Furthermore, participants who completed LTFU were 6.77 months younger at pretreatment than dropouts. Although this difference is small, it could have reflected confounding factors (eg, dropouts having more responsibilities/less availability that might come with age) and should be investigated in future research. Also, there were more participants identifying as Asian in TranS-C than in PE at LTFU. Some evidence suggests that adults who are Asian American tend to experience differences in sleep functioning compared with Black or White American adults, which could be impacted by systemic discrimination, cultural attitudes toward sleep, and poor mental health.^51^ In the LTFU, however, the differences could have occurred by chance, given that participants were randomly assigned at pretreatment, and the number of participants identifying as Asian from the original RCT who did not participate in LTFU was not significantly different between the 2 conditions.

Second, a preregistered aim of this study was to compare the effects of TranS-C with PE on sleep and circadian functioning, health risk, sleep health behavior, and habits at LTFU. This has the downside of necessitating a range of primary outcomes and multiple comparisons that increase risk of type I error. As such, replication of these findings with fewer primary outcomes may be valuable. Third, the generalizability of results to youth in low-income families should be tested, especially because our sample lacked diversity in income—and some evidence suggests that youth of low socioeconomic status are more likely to show a waning of treatment effects.^52^ Fourth, although the use of EMA enhances the ecological validity of the findings, our use of binary outcome measures for social health (whether anyone was with them when completing the questionnaire) and physical health (whether they were physically active that day) could have reduced granularity in these outcomes and increased risk of type II error. Indeed, self-reported social and physical health risk were associated with poorer sleep functioning, whereas EMA social and physical health risk were not associated with any sleep or circadian outcome. Future research may consider more granular EMA measures of social health (eg, “How connected do you feel with them?”) and physical health (eg, “How many minutes did you engage in moderate to vigorous exercise today?”).

In sum, one goal of EBPTs is to ameliorate the targets contributing to psychological problems by fostering health-promoting behavior.^1^ However, what happens if the key target would have resolved without intervention? Consistent with prior research,^9,10,12^ we found that despite initial gains and maintenance of effects (reduced eveningness and wakeup variability),^23,25,26^ a fade-out in differences between TranS-C and PE occurred when assessed approximately 8 years after intervention. Again, this is a troubling finding because, as in prior studies,^38^ sleep and circadian problems were associated with poorer health risk across multiple domains. Although many TranS-C and PE participants appeared to have outgrown their eveningness with age, many did not (Table 1). There remains a need to understand the effects of and processes contributing to the remaining young adults exhibiting eveningness.

Our findings raise the question of whether there may be a modifiable contributor to eveningness chronotype that can be leveraged in future iterations of TranS-C and similar interventions. One possibility is sleep health behavior change. In the context of youth psychological treatment, cultivating long-term behavior change in the face of emerging vulnerabilities and impending developmental transitions adds increasing complexity to the puzzle. Nevertheless, exploring how to scaffold the process of forging long-term maintenance plans and anticipating future obstacles will surely be fruitful. As habits predict future behavior,^40^ applying the science of habit formation to augment behavior change presents an exciting avenue of research to improve TranS-C and perhaps psychological treatments more broadly.^1,55^

## Supplementary Material

Supplemental Material

## Figures and Tables

**TABLE 1:** Demographic Variables and Participant Characteristics

Characteristic	Whole sample (N = 106)		TranS-C (n = 51)		PE (n = 55)		TranS-C vs PE difference
	n	%	n	%	n	%	*p*
Assigned female at birth^a^	62	58.49	29	56.86	33	60.00	.743
Hispanic/Latine	13	12.26	8	15.69	5	9.09	.301
Preferred not to answer	1	0.94	1	1.96	0	0.00	.297
Race^b^							
American Indian/Alaskan Native	0	0.00	0	0.00	0	0.00	—
Asian	16	15.09	12	23.53	4	7.27	**.019**
Black	14	13.21	4	7.84	10	18.18	.116
Native Hawaiian or Pacific Islander	1	0.94	1	1.96	0	0.00	.297
Not listed	6	5.66	3	5.88	3	5.45	.924
More than one race	15	14.15	7	13.73	8	14.55	.904
White	71	66.98	33	64.71	38	69.09	.632
Family annual income at pretreatment^c^							
≤$20,000	3	2.83	1	1.96	2	3.64	.172
$20,001-$50,000	15	14.15	7	13.73	8	14.55	.904
$50,001-$100,000	22	20.75	14	27.45	8	14.55	.102
≥$100,000	64	60.38	28	54.90	36	65.45	.267
Preferred not to answer	2	1.89	1	1.96	1	1.81	.955
Level of education^d^							
Without high school diploma	3	2.83	3	5.88	0	0.00	.068
High school graduate	12	11.32	8	15.69	4	7.27	.172
Some college education	64	60.38	29	56.86	35	63.64	.476
Degree from 4-y college or more	27	25.47	11	21.57	16	29.09	.375
	**Mean**	**SD**	**Mean**	**SD**	**Mean**	**SD**	***p***
Age at pretreatment^e^	14.52	1.73	14.57	1.87	14.47	1.60	.768
Age at LTFU^f^	22.45	1.94	22.52	1.95	22.39	1.94	.731
	**n**	**%**	**n**	**%**	**n**	**%**	***p***
Text condition assignment							
No text	47	44.34	20	39.22	27	49.09	.307
Push text	29	27.36	18	35.29	11	20.00	.078
Pull text	30	28.30	13	25.49	17	30.91	.536
Sleep Health Composite dimensions at LTFU^g^							
Regularity	28	26.42	14	27.45	14	25.45	.815
Satisfaction	9	8.49	6	11.76	3	5.45	.244
Alertness	20	18.87	11	21.57	9	16.36	.493
Timing	45	42.45	23	45.10	22	40.00	.596
Efficiency	75	70.75	32	62.75	43	78.18	.081
Duration	45	42.45	22	43.14	23	41.82	.743
Family annual income at LTFU^h^							
≤$20,000	2	1.89	2	3.92	0	0	.138
$20,001-$50,000	8	7.55	4	7.84	4	7.27	.912
$50,001-$100,000	34	32.08	16	31.37	18	32.73	.881
≥$100,000	58	54.72	26	50.98	32	58.18	.457
Personal annual income at LTFU^i^							
≤$20,000	74	69.81	37	72.55	37	67.27	.554
$20,001-$50,000	24	22.64	11	21.57	13	23.64	.800
$50,001-$100,000	6	5.66	3	5.88	3	5.45	.924
≥$100,000	2	1.89	0	0.00	2	3.64	.169
Participants exhibiting a tendency toward eveningness^j^							
Eveningness, lowest quartile of CSM ≤28	46	43.4	27	52.94	19	34.55	.056

Note: Boldface indicates significant (p < .05) results. CSM = Composite Scale of Morningness; LTFU = long-term follow-up; PE = psychoeducation; TranS-C = Transdiagnostic Sleep and Circadian Intervention.

a Participants assigned the biological sex of female at birth.

b Participant’s race.

c Participants’ family yearly income in US dollars when they began treatment.

d Participants’ level of education at LTFU.

e Participants’ age when they began treatment.

f Participants’ age when they completed LTFU.

g Participants’ categorization into poor (as opposed to healthy) in the components of the Sleep Health Composite.^38^

h Participants’ family yearly income in US dollars at LTFU.

i Participants’ personal yearly income in US dollars at LTFU.

j Number and percentage of participants scoring in the lowest quartile of CSM. A score of 28 or below indicates a tendency toward eveningness.^53,54^

**TABLE 2:** Regression Analysis Results of Primary, Secondary, and Other Outcomes Contrasting Transdiagnostic Sleep and Circadian Intervention (TranS-C) vs Psychoeducation (PE) at Long-Term Follow-Up (LTFU)

	*d*	95% CI	*p*
Primary outcomes			
CSM	−0.12	[−0.50, 0.27]	.552
PROMIS-SRI	−0.08	[−0.46, 0.31]	.700
PROMIS-SD	−0.17	[−0.55, 0.22]	.393
Emotional health risk	0.21	[−0.17, 0.59]	.283
Cognitive health risk	−0.11	[−0.49, 0.27]	.572
Behavioral health risk	−0.11	[−0.49, 0.27]	.561
Social health risk	−0.04	[−0.42, 0.34]	.840
Physical health risk	−0.04	[−0.42, 0.34]	.840
Utilization of sleep health behaviors	−0.21	[−0.59, 0.17]	.276
Habit formation	−0.22	[−0.60, 0.16]	.255
Secondary outcomes			
PSQI	−0.2	[−0.59, 0.17]	.276
Midpoint fluctuation	−0.22	[−0.60, 0.16]	.255
Sleep Health Composite	0.18	[−0.20, 0.56]	.357
	**β**	**95% CI**	***p***
EMA outcomes			
Emotional health risk	−.20	[−0.59, 0.19]	.308
Cognitive health risk	−.12	[−0.52, 0.28]	.559
Behavioral health risk	−.08	[−0.49, 0.33]	.698
	**OR**	**95% CI**	***p***
Social health risk	1.26	[0.57, 2.82]	.566
Physical health risk	0.88	[0.39, 1.99]	.754
Other outcomes	***d***	**95% CI**	***p***
BMI	0.17	[−0.21, 0.55]	.391
Waist circumference	0.01	[−0.37, 0.39]	.952

Note: We covaried participants’ sex assigned at birth, age at long-term follow-up, and text messaging intervention. BMI = body mass index; CSM = Composite Scale of Morningness; EMA = ecological momentary assessment; OR = odds ratio; PROMIS-SD = Patient-Reported Outcomes Measurement Information System–Sleep Disturbance; PROMIS-SRI = PROMIS–Sleep-Related Impairment; PSQI = Pittsburgh Sleep Quality Index.

**TABLE 3:** Regression Analyses of Associations Between Health Risk and Sleep and Circadian Functioning at Long-Term Follow-Up (LTFU)

Self-report health risk	β	95% CI	*p*
Emotional health risk			
CSM	**−.27**	**[−0.46, −0.09]**	**.004**
PROMIS-SRI	**.60**	**[0.45, 0.75]**	**<.001**
PROMIS-SD	**.50**	**[0.33, 0.66]**	**<.001**
PSQI	**.42**	**[0.25, 0.59]**	**<.001**
Midpoint fluctuation	−.05	[−0.26, 0.15]	.601
Sleep Health Composite	**−.34**	**[−.52, −.16]**	**<.001**
Cognitive health risk			
CSM	**−.22**	**[−0.42, −0.02]**	**.029**
PROMIS-SRI	**.55**	**[0.39, 0.72]**	**<.001**
PROMIS-SD	**−.34**	**[−0.52, −0.16]**	**<.001**
PSQI	**.43**	**[0.24, 0.61]**	**<.001**
Midpoint fluctuation	**.37**	**[0.18, 0.55]**	**<.001**
Sleep Health Composite	−.01	[−0.22, 0.21]	.946
Behavioral health risk			
CSM	**−.24**	**[−0.43, −0.04]**	**.019**
PROMIS-SRI	−.01	[−0.21, 0.19]	.930
PROMIS-SD	**.22**	**[0.03, 0.42]**	**.022**
PSQI	**.26**	**[0.07, 0.45]**	**.009**
Midpoint fluctuation	**.35**	**[0.17, 0.54]**	**<.001**
Sleep Health Composite	.01	[−0.20, 0.22]	.913
Social health risk			
CSM	−.14	[−0.33, 0.06]	.169
PROMIS-SRI	−.05	[−0.24, 0.15]	.636
PROMIS-SD	**.21**	**[0.02, 0.40]**	**.029**
PSQI	**.26**	**[0.07, 0.45]**	**.007**
Midpoint fluctuation	.16	[−0.03, 0.35]	.098
Sleep Health Composite	−.02	[−0.22, 0.18]	.853
Physical health risk			
CSM	−.14	[−0.33, 0.05]	.147
PROMIS-SRI	−.01	[−0.21, 0.19]	.917
PROMIS-SD	**.32**	**[0.14, 0.50]**	**.001**
PSQI	**.23**	**[0.04, 0.42]**	**.019**
Midpoint fluctuation	.12	[−0.07, 0.31]	.207
Sleep Health Composite	−.05	[−.26, 0.15]	.608
**EMA health risk**	**β**	**95% CI**	***p***
Emotional health risk			
CSM	−.14	[−0.34, 0.05]	.151
PROMIS-SRI	**.22**	**[0.03, 0.41]**	**.027**
PROMIS-SD	**.21**	**[0.01, 0.40]**	**.040**
PSQI	**.21**	**[0.02, 0.40]**	**.034**
Midpoint fluctuation	−.13	[−0.33, 0.08]	.217
Sleep Health Composite	−.17	[−0.37, 0.02]	.081
Cognitive health risk			
CSM	−.12	[−0.32, 0.08]	.247
PROMIS-SRI	**.37**	**[0.18, 0.56]**	**<.001**
PROMIS-SD	.17	[−0.03, 0.38]	.097
PSQI	.15	[−0.05, 0.35]	.139
Midpoint fluctuation	−.20	[−0.40, 0.01]	.067
Sleep Health Composite	−.14	[−0.34, 0.06]	.174
Behavioral health risk			
CSM	−.04	[−0.24, 0.17]	.723
PROMIS-SRI	.09	[−0.11, 0.30]	.362
PROMIS-SD	.19	[−0.02, 0.40]	.070
PSQI	**.36**	**[0.17, 0.55]**	**<.001**
Midpoint fluctuation	.01	[−0.20, 0.22]	.933
Sleep Health Composite	**−.20**	**[−0.40, −0.00]**	**.047**
	**OR**	**95% CI**	***p***
Social health risk			
CSM	0.97	[0.64, 1.47]	.879
PROMIS-SRI	1.14	[0.75, 1.72]	.548
PROMIS-SD	1.35	[0.87, 2.09]	.183
PSQI	1.09	[0.72, 1.64]	.691
Midpoint fluctuation	1.22	[0.79, 1.90]	.367
Sleep Health Composite	1.03	[0.68, 1.54]	.903
Physical health risk			
CSM	0.83	[0.55, 1.25]	.378
PROMIS-SRI	1.04	[0.69, 1.55]	.852
PROMIS-SD	0.80	[0.52, 1.23]	.313
PSQI	1.08	[0.72, 1.63]	.699
Midpoint fluctuation	0.80	[0.52, 1.24]	.321
Sleep Health Composite	1.06	[0.71, 1.58]	.776

Note: We covaried treatment (TranS-C vs PE), participants’ sex assigned at birth, age at LTFU, and text messaging intervention. Boldface indicates significant (p < .05) results. CSM = Composite Scale of Morningness; OR = odds ratio; PE = Psychoeducation; PROMIS-SD = Patient-Reported Outcomes Measurement Information System–Sleep Disturbance; PROMIS-SRI = PROMIS–Sleep-Related Impairment; PSQI = Pittsburgh Sleep Quality Index; TranS-C = Transdiagnostic Sleep and Circadian Intervention.

**TABLE 4:** Associations Between Utilization and Habit Formation of Sleep Health Behaviors With Sleep and Circadian Functioning at Long-Term Follow-Up (LTFU)

	β	95% CI	* p*
Utilization			
CSM	**.23**	**[0.03, 0.42]**	** .023**
PROMIS-SRI	.00	[−0.20, 0.20]	.981
PROMIS-SD	.06	[−0.14, 0.26]	.575
PSQI	−.02	[−0.23, 0.18]	.811
Midpoint fluctuation	.01	[−0.20, 0.23]	.907
Sleep Health Composite	.02	[−0.19, 0.22]	.883
Habit formation			
CSM	**.23**	**[0.03, 0.43]**	** .025**
PROMIS-SRI	.00	[−0.20, 0.21]	.966
PROMIS-SD	.02	[−0.19, 0.22]	.858
PSQI	−.09	[−0.29, 0.12]	.410
Midpoint fluctuation	−.08	[−0.30, 0.15]	.500
Sleep Health Composite	.05	[−0.16, 0.26]	.645

Note: We covaried treatment (TranS-C vs PE), participants’ sex assigned at birth, age at LTFU, and text messaging intervention. Boldface indicates significant (p < .05) results. CSM = Composite Scale of Morningness; PE = psychoeducation; PROMIS-SD = Patient-Reported Outcomes Measurement Information System–Sleep Disturbance; PROMIS-SRI = PROMIS–Sleep-Related Impairment; PSQI = Pittsburgh Sleep Quality Index; TranS-C = Transdiagnostic Sleep and Circadian Intervention.

## Diversity & Inclusion Statement:

We worked to ensure race, ethnic, and/or other types of diversity in the recruitment of human participants. We worked to ensure that the study questionnaires were prepared in an inclusive way. We worked to ensure sex and gender balance in the recruitment of human participants. One or more of the authors of this paper self-identifies as a member of one or more historically underrepresented racial and/or ethnic groups in science. One or more of the authors of this paper self-identifies as a member of one or more historically underrepresented sexual and/or gender groups in science. One or more of the authors of this paper self-identifies as living with a disability. One or more of the authors of this paper received support from a program designed to increase minority representation in science.

## References

[1] Harvey AG, Callaway CA, Zieve GG, Gumport NB, Armstrong CC. Applying the science of habit formation to evidence-based psychological treatments for mental illness. Perspect Psychol Sci 2022;17(2):572–589. 10.1177/1745691621995752 34495781 PMC12318445

[2] Kazdin AE. Innovations in Psychosocial Interventions and Their Delivery: Leveraging Cutting-Edge Science to Improve the World’s Mental Health. Oxford: Oxford University Press; 2018.

[3] Clark DM. Realizing the mass public benefit of evidence-based psychological therapies: the IAPT program. Annu Rev Clin Psychol 2018;14:159–183. 10.1146/annurev-clinpsy-050817-084833 29350997 PMC5942544

[4] Weisz JR, McCarty CA, Valeri SM. Effects of psychotherapy for depression in children and adolescents: a meta-analysis. Psychol Bull. 2006;132(1):132–149. 10.1037/0033-2909.132.1.132 16435960 PMC2150594

[5] Ginsburg GS. The Child Anxiety Prevention Study: intervention model and primary outcomes. J Consult Clin Psychol 2009;77(3):580. 19485597 10.1037/a0014486 PMC3373966

[6] Jernelöv S, Blom K, Hentati Isacsson N, Very long-term outcome of cognitive behavioral therapy for insomnia: one- and ten-year follow-up of a randomized controlled trial. Cogn Behav Ther 2022;51(1):72–88. 10.1080/16506073.2021.2009019 35099359

[7] Bell EC, Marcus DK, Goodlad JK. Are the parts as good as the whole? A meta-analysis of component treatment studies. J Consult Clin Psychol 2013;81(4):722. 23688145 10.1037/a0033004

[8] Bailey D, Duncan GJ, Odgers CL, Yu W. Persistence and fadeout in the impacts of child and adolescent interventions. J Res Educ Eff 2017;10(1):7–39. 10.1080/19345747.2016.1232459 29371909 PMC5779101

[9] Espil FM, Woods DW, Specht MW, Long-term outcomes of behavior therapy for youth with Tourette disorder. J Am Acad Child Adolesc Psychiatry. 2022;61(6): 764–771. 10.1016/j.jaac.2021.08.022 34508805

[10] Scott S, Briskman J, O’Connor TG. Early prevention of antisocial personality: long-term follow-up of two randomized controlled trials comparing indicated and selective approaches. Am J Psychiatry. 2014;171(6):649–657. 10.1176/appi.ajp.2014.13050697 24626738

[11] Gumport NB, Dolsen MR, Harvey AG. Usefulness and utilization of treatment elements from the Transdiagnostic Sleep and Circadian Intervention for adolescents with an evening circadian preference. Behav Res Ther 2019;123:103504. 31678861 10.1016/j.brat.2019.103504 PMC6864305

[12] Bertholet N, Studer J, Cunningham JA, Gmel G, Burnand B, Daeppen JB. Four-year follow-up of an internet-based brief intervention for unhealthy alcohol use in young men. Addiction. 2018;113(8):1517–1521. 10.1111/add.14179 29396897

[13] Hasler BP. Chronotype and mental health: timing seems to matter, but how, why, and for whom? World Psychiatry. 2023;22(2):329–330. 10.1002/wps.21092 37159371 PMC10168154

[14] Merikanto I, Partonen T. Increase in eveningness and insufficient sleep among adults in population-based cross-sections from 2007 to 2017. Sleep Med 2020;75:368–379. 10.1016/j.sleep.2020.07.046 32950882

[15] Crowley SJ, Acebo C, Carskadon MA. Sleep, circadian rhythms, and delayed phase in adolescence. Sleep Med 2007;8(6):602–612. 10.1016/j.sleep.2006.12.002 17383934

[16] Kramer Fiala Machado A, Wendt A, Baptista Menezes AM, Gonçalves H, Wehrmeister FC. Sleep duration trajectories from adolescence to emerging adulthood: Findings from a population-based birth cohort. J Sleep Res 2021;30(3):e13155. 10.1111/jsr.13155 32808393

[17] McGlinchey EL, Harvey AG. Risk behaviors and negative health outcomes for adolescents with late bedtimes. J Youth Adolesc 2015;44(2):478–488. 24599733 10.1007/s10964-014-0110-2 PMC4164586

[18] Azad-Marzabadi E, Amiri S. Morningness-eveningness and emotion dysregulation incremental validity in predicting social anxiety dimensions. Int J Gen Med 2017;10: 275–279. 10.2147/ijgm.S144376 28919804 PMC5593450

[19] Randler C, Frech D. Correlation between morningness–eveningness and final school leaving exams. Biol Rhythm Res 2006;37(3):233–239. 10.1080/09291010600645780

[20] Digdon NL, Howell AJ. College students who have an eveningness preference report lower self-control and greater procrastination. Chronobiol Int 2008;26:1029–1046. 10.1080/07420520802553671 19005903

[21] Randler C Association between morningness–eveningness and mental and physical health in adolescents. Psychol Health Med 2011;16(1):29–38. 10.1080/13548506.2010.521564 21218362

[22] Tavernier R, Willoughby T. A longitudinal examination of the bidirectional association between sleep problems and social ties at university: the mediating role of emotion regulation. J Youth Adolesc 2015;44(2):317–330. 10.1007/s10964-014-0107-x 24578222

[23] Harvey AG, Hein K, Dolsen MR, Modifying the impact of eveningness chronotype (“night-owls”) in youth: a randomized controlled trial. J Am Acad Child Adolesc Psychiatry. 2018;57(10):742–754. 30274649 10.1016/j.jaac.2018.04.020 PMC6923796

[24] Harvey AG, Buysse DJ. Treating Sleep Problems: A Transdiagnostic Approach. New York, NY: Guilford Press; 2017.

[25] Dolsen EA, Dong L, Harvey AG. Transdiagnostic sleep and circadian intervention for adolescents plus text messaging: randomized controlled trial 12-month follow-up. J Clin Child Adolesc Psycho 2023;52(6):750–762. 10.1080/15374416.2021.1978295 PMC9213566 34936528

[26] Dong L, Dolsen MR, Martinez A, Notsu H, Harvey AG. A transdiagnostic sleep and circadian intervention for adolescents: six-month follow-up of a randomized controlled trial. J Child Psychol Psychiatry. 2020;61:653–661. 31773734 10.1111/jcpp.13154 PMC7242125

[27] Blake M, Schwartz O, Waloszek JM, The SENSE study: Treatment mechanisms of a cognitive behavioral and mindfulness-based group sleep improvement intervention for at-risk adolescents. Sleep. 2017;40(6). 10.1093/sleep/zsx061 28431122

[28] de Bruin EJ, Bögels SM, Oort FJ, Meijer AM. Efficacy of cognitive behavioral therapy for insomnia in adolescents: a randomized controlled trial with internet therapy, group therapy and a waiting list condition. Sleep. 2015;38(12):1913–1926. 26158889 10.5665/sleep.5240 PMC4667374

[29] Hasler BP, Buysse DJ, Germain A. Shifts toward morningness during behavioral sleep interventions are associated with improvements in depression, positive affect, and sleep quality. Behav Sleep Med 2016;14(6):624–635. 26549156 10.1080/15402002.2015.1048452 PMC4867300

[30] Campbell IG, Cruz-Basilio A, Figueroa JG, Bottom VB. Earlier bedtime and its effect on adolescent sleep duration. Pediatrics. 2023 1;152(1):e2022060607. 10.1542/peds.2022-060607 37305962 PMC10312236

[31] Harvey AG, Soehner AM, Kaplan KA, Treating insomnia improves sleep, mood and functioning in bipolar disorder: A pilot randomized controlled trial. J Consult Clin Psychol 2015;83(3):564–577. 25622197 10.1037/a0038655 PMC4446240

[32] Randler C Validation of the full and reduced Composite Scale of Morningness. Biol Rhythm Res 2009;40(5):413–423. 10.1080/09291010902731213

[33] Yu L, Buysse DJ, Germain A, Development of short forms from the PROMIS Sleep Disturbance and Sleep-Related Impairment item banks. Behav Sleep Med 2011;10(1): 6–24. 10.1080/15402002.2012.636266 22250775 PMC3261577

[34] HealthMeasures. 2024. Accessed March 22, 2024. https://www.healthmeasures.net/

[35] Bei B, Wiley JF, Trinder J, Manber R. Beyond the mean: a systematic review on the correlates of daily intraindividual variability of sleep/wake patterns. Sleep Med Rev 2016; 28:108–124. 10.1016/j.smrv.2015.06.003 26588182

[36] Johnson NL, Kirchner HL, Rosen CL, Sleep estimation using wrist actigraphy in adolescents with and without sleep disordered breathing: a comparison of three data modes. Sleep. 2007;30:899–905. 17682661 10.1093/sleep/30.7.899 PMC1978368

[37] Buysse DJ, Cheng Y, Germain A, Night-to-night sleep variability in older adults with and without chronic insomnia. Sleep Med 2010;11(1):56–64. 19962939 10.1016/j.sleep.2009.02.010 PMC2818595

[38] Dong L, Martinez AJ, Buysse DJ, Harvey AG. A composite measure of sleep health predicts concurrent mental and physical health outcomes in adolescents prone to eveningness. Sleep Health. 2019;5(2):166–174. 10.1016/j.sleh.2018.11.009 30928117 PMC6452900

[39] Burchinal M, Roberts J, Hooper S, Zeisel S. Cumulative risk and early cognitive development: a comparison of statistical risk models. Dev Psychol 2000;36(6):793–807. 10.1037//0012-1649.36.6.793 11081702

[40] Gardner B, Abraham C, Lally P, de Bruijn GJ. Towards parsimony in habit measurement: Testing the convergent and predictive validity of an automaticity subscale of the Self-Report Habit Index. Int J Behav Nutr Phys Act 2012;9(1):102. 22935297 10.1186/1479-5868-9-102 PMC3552971

[41] Chin R, Lee BY. Assessing data quality and transforming data. In: Chin R, Lee BY, eds. Principles and Practice of Clinical Trial Medicine. Cambridge, MA: Academic Press; 2008:303–323.

[42] Nakagawa S, Cuthill IC. Effect size, confidence interval and statistical significance: a practical guide for biologists. Biol Rev 2007;82(4):591–605. 10.1111/j.1469-185X.2007.00027.x 17944619

[43] Randler C, Faßl C, Kalb N. From Lark to Owl: developmental changes in morningness-eveningness from new-borns to early adulthood. Sci Rep 2017;7(1):45874. 10.1038/srep45874 28378787 PMC5381104

[44] Roenneberg T, Kuehnle T, Pramstaller PP, A marker for the end of adolescence. Curr Biol 2004;14(24):R1038–R1039. 10.1016/j.cub.2004.11.039 15620633

[45] Carskadon MA, Vieira C, Acebo C. Association between puberty and delayed phase preference. Sleep. 1993;16(3):258–262. 8506460 10.1093/sleep/16.3.258

[46] Garland SN, Carlson LE, Stephens AJ, Antle MC, Samuels C, Campbell TS. Mindfulness-based stress reduction compared with cognitive behavioral therapy for the treatment of insomnia comorbid with cancer: a randomized, partially blinded, non-inferiority trial. J Clin Oncol 2014;32(5):449–457. 10.1200/JCO.2012.47.7265 24395850

[47] Grossman P, Tiefenthaler-Gilmer U, Raysz A, Kesper U. Mindfulness training as an intervention for fibromyalgia: evidence of postintervention and 3-year follow-up benefits in well-being. Psychother Psychosom 2007;76(4):226–233. 10.1159/000101501 17570961

[48] Sarfan LD, Zieve GG, Mujir F, Gumport NB, Xiong M, Harvey AG. Serial mediators of memory support strategies used with cognitive therapy for depression: improving outcomes through patient adherence and treatment skills. Behav Ther 2023;54(1):141–155. 10.1016/j.beth.2022.07.012 36608972 PMC10927275

[49] Pfeifer JH, Berkman ET. The development of self and identity in adolescence: neural evidence and implications for a value-based choice perspective on motivated behavior. Child Dev Perspect 2018;12(3):158–164. 10.1111/cdep.12279 31363361 PMC6667174

[50] Karlson CW, Rapoff MA. Attrition in randomized controlled trials for pediatric chronic conditions. J Pediatr Psychol 2009;34(7):782–793. 10.1093/jpepsy/jsn122 19064607

[51] Nandagiri V, Vannemreddy S, Spector A. Sleep disparities in Asian Americans: a comprehensive review. J Clin Sleep Med 2023;19(2):393–402. 10.5664/jcsm.10330 36239044 PMC9892749

[52] Eyberg SM, Edwards D, Boggs SR, Foote R. Maintaining the treatment effects of parent training: The role of booster sessions and other maintenance strategies. Clinical Psychology: Science and Practice. 1998;5(4):544–554. 10.1111/j.1468-2850.1998.tb00173.x

[53] Monk TH, Buysse DJ, Potts JM, DeGrazia JM, Kupfer DJ. Morningness-eveningness and lifestyle regularity. Chronobiol Int 2004;21(3):435–443. 10.1081/CBI-120038614 15332448

[54] Škvorc L, Bjelajac AK. Sleep beliefs and circadian typology of helping professions students. International Online Journal of Educational Sciences. 2016;8(5):69–78. 10.15345/iojes.2016.05.008

[55] Susman ES, Chen S, Kring AM, Harvey AG. Daily micropractice can augment single-session interventions: a randomized controlled trial of self-compassionate touch and examining their associations with habit formation in US college students. Behav Res and Ther 2024;175:104498. 10.1016/j.brat.2024.104498 38412573

