# Supplementary material for: Transdiagnostic Sleep and Circadian Intervention in Youth: Long-Term Follow-Up of a Randomized Controlled Trial
Source: J Am Acad Child Adolesc Psychiatry. Author manuscript; Available in PMC 2025 Aug 3. (PMC12318444; doi:10.1016/j.jaac.2024.05.001)
Supplement: Supplemental Material [file NIHMS2094800-supplement-Supplemental_Material.docx]

**Supplement 1**

**Supplemental Methods**

**Participants and Procedures**

**Inclusion and Exclusion Criteria of the Original RCT.**

As detailed in the manuscript of the original RCT,^1^ the inclusion and exclusion criteria for the parent study were as follows:

Individuals were eligible if they (a) were between 10 and 18 years old, living with a parent or guardian, and attending a class/job by 9 am at least 3 days per week; (b) were fluent in English; (c) were able and willing to give informed assent; and (d) reported eveningness as demonstrated by scoring within the lowest quartile of the Children’s Morningness–Eveningness Preferences Scale (CMEP; 27 or lower) and had a 7-day sleep diary showing a sleep onset time of 10:40 pm or later for 10- to 13-year- olds,^2,3^ 11:00 pm or later for 14- to 16-year-olds, and 11:20 pm or later for 17- to 18-year-olds at least 3 nights per week. In addition, this sleep pattern had to have been present for the past 3 months. Finally, participants had to fall into an “at-risk” range on measures of at least one of the five health domains (Table S2).

Exclusion criteria were (a) an active, progressive physical illness or neurological degenerative disease directly related to the onset and course of the sleep disturbance; (b) evidence of obstructive sleep apnea, restless legs syndrome, or periodic limb movement disorder (youth presenting with provisional diagnoses of any of these disorders were referred for a nonstudy polysomnography evaluation at the parent’s discretion and were enrolled only if the diagnosis was disconfirmed); (c) significantly impairing pervasive developmental disorder; (d) bipolar disorder, schizophrenia, or another current Axis I disorder if there was a risk of harm if treatment were delayed. Participants ceased taking medications that alter sleep (e.g., hypnotics) 4 weeks prior to the assessment (2 weeks for melatonin) or were excluded. Finally, history of substance dependence in the past 6 months or current suicide risk sufficient to preclude treatment on an outpatient basis was exclusionary.

In other words, receipt of another sleep treatment was the only type of treatment excluded. We wanted to be sure that any improvements in sleep could be attributed to TranS-C or PE, not the other sleep treatment. Other medications were allowed. A medication-free group would have been nonrepresentative. We also included youth with Axis I psychiatric comorbidity (except for bipolar disorder and schizophrenia), even if they were receiving treatment for that comorbidity.

**Sleep and Circadian Outcomes**

**Derivation of the Sleep Health Composite.** Derivation of the Sleep Health Composite was based on several considerations. We dichotomized each dimension as “good” or “poor”. This approach is more feasible and deployable in clinical practice and has been documented in prior studies^4^. We coded the Sleep Health Composite as 1 = good and 0 = poor, with scores ranging from 0 to 6; higher scores indicated better sleep health.^4^ We determined the cut-off point for each dimension based on studies in young adults and adults.^5–7^ The cut-off points for the six dimensions were Regularity (Standard deviation of the midpoint of sleep in participants’ sleep diary ≤ 1 hour), satisfaction (Sleep quality question on PROMIS-SD ≤ 3); alertness (Daytime sleepiness question on PROMIS-SRI ≤ 3); timing (2:00 am ≤ midpoint of sleep in sleep diary ≤ 4:00 am), efficiency (≥ 85% in the sleep diary); duration (7–9 hours of sleep in sleep diary). Participants completed sleep diaries completed for one week. We used an adapted version of the consensus sleep diary.^8–10^

**The Five Health-Relevant Domains**

**Derivation of the Self-Report Composite Risk Score.** We used five composite risk scores as primary outcomes, composed of measures of emotional, cognitive, behavioral, social, and physical health, to indicate functioning in these five domains. For each of the five health domains, we calculated composite risk scores by standardizing the raw score (i.e., z-scoring) for each measure and then averaging the standardized scores from each respective health domain’s measure. In line with prior research,^4^ we reverse-coded summary scores for certain measures when appropriate such that all scores of the measures within a domain indicate the same direction (i.e., high scores indicate greater risk). Specific measures for each domain are listed below.

***Emotional Health.*** The 21-item version of the Depression, Anxiety, and Stress Scales (DASS-21) was administered^11^. Participants responded to 21 items on a 4-point scale.

***Cognitive Health.*** A composite score of the 6-item Patient-Reported Outcomes Measurement Information System-Cognitive Function (PROMIS-CF; e.g., “in the past 7 days… my thinking has been slow”),^12^ and the Patient-Reported Outcomes Measurement Information System-Cognitive Function - Abilities (PROMIS-CF-A; e.g., “in the past 7 days… I have been able to concentrate”)^13^ was used to assess functioning in the Cognitive domain based on a 5-point rating scale.

***Behavioral Health.*** A composite score of the 8-item Brief Sensation Seeking Scale (BSSS),^14,15^ the 7-item Patient-Reported Outcomes Measurement Information System-Alcohol Use (PROMIS-AU),^16^ and the 7-item Patient-Reported Outcomes Measurement Information System-Severity of Substance Use was used to assess functioning in the Behavioral domain (PROMIS-SSU; Pilkonis et al., 2015).^17^ Each of these measures used a 5-point rating scale.

***Social Health.*** A composite score of the Patient-Reported Outcomes Measurement Information System-Companionship (PROMIS-C; e.g., “Do you have someone with whom to have fun”),^18,19^ Patient-Reported Outcomes Measurement Information System-Emotional Support (PROMIS-ES “I have someone who will listen to me when I need to talk”),^18^ Patient-Reported Outcomes Measurement Information System-Social Isolation (PROMIS-SI; e.g., “I feel left out”),^18,19^ and Patient-Reported Outcomes Measurement Information System-Ability to Participate (PROMIS-AP; e.g., “I have trouble doing all of the family activities that I want to do”),^18–20^ was used to assess functioning in the Social domain.^18–20^ Each of these questionnaires had four items and was rated on a five-point scale.

***Physical Health.*** A composite of two scales: (1) The Modifiable Activity Questionnaire measures self-reported past-month participation in leisure/sports activities (MAQ).^21^ This was calculated as the number of hours in the last month spent being active or exercising, where higher scores = greater numbers of active hours. (2) The Physical Health Questionnaire-15 (PHQ-15)^22–24^ measures physical health symptoms (i.e., somatic problems such as stomach pain, back pain, headaches, dizziness, or shortness of breath) over the past month using 15 items on a 0 to 2 rating scale.

**Derivation of Ecological Momentary Assessment (EMA) Composite Risk Score.**  EMA was collected by sending a Qualtrics link via text message 2x per day on weekdays (after waking and in the evening) and 4x per day on weekends (one after waking, one in the evening, and the other two at random times in between). The questions assess the five health-relevant domains. We used a random number generator to determine the exact times the texts would be sent. The first text of the day was a time randomized between 30 to 90 minutes after their latest wake-up time. The final text of the day was a randomized time between one to three hours before their earliest bedtime. On weekends, the second text of the day was a randomized time between four and six hours after awakening, and the third text of the day was a randomized time between eight and ten hours after awakening. If no response was received within 1 hour, a reminder was sent. The sleep diary was also completed via a Qualtrics link that was sent within 5 minutes of the participant's latest awakening. If no response was received within 1 hour, a reminder was sent. We used five composite risk scores, composed of ecological momentary assessments of emotional, cognitive, behavioral, social, and physical health, to indicate functioning in these five domains. For each of the five health domains, we calculate composited risk scores in the same manner as described in the section on “Health-relevant domains.” The questions are adapted from prior research.^1,25^ Specific measures for each domain are listed below:

***Emotional Health.*** As an index of emotional experience, a short version of the Positive and Negative Affect Schedule was administered.^26^ The Positivity Ratio^27,28^ was used to drive an index of emotional health.

***Cognitive Health*.** Concentration, distractedness, and focus are rated on a 5-point scale.^1,25^

***Behavioral Health.*** We assessed eating, caffeine, alcohol, and nicotine, and participants were also asked, “Did you use drugs other than alcohol and your prescribed medication? (e.g., cocaine, heroin, acid, Xanax, Vicodin)”.^29,30^ To weigh the relative risk of drugs and alcohol in relation to junk food, we used the economic costs of drugs and alcohol to society in the United States relative to poor nutrition to calculate a weighted sum score (i.e., $943.1 billion for drugs and alcohol,^31^ divided by $217.7 billion for obesity,^32,33^ adjusted for inflation^34^ is 4.33). Thus, behavioral health was calculated by taking the sum of one’s daily intake of drugs and alcohol (multiplied by 4.33) and junk food behavior. There were only three drugs that participants noted using in our sample: alcohol, tobacco, and cannabis. We weighted alcohol and drugs by their average level of harm relative to each other as assessed by two studies,^35,36^ one of which^36^ largely replicated the results of the prior study^35^ (Cannabis = 1.095, Tobacco/Nicotine = 1.75, Alcohol = 2.88).

At each text, participants reported and self-coded their current and most recent meal’s intake of the following: a. Fresh fruits, b. Fresh vegetables, c. Protein (meats, eggs, nuts, seeds, meat alternatives), d. Dairy products (cheese, milk), e. Grains (rice, pasta, bread), f. Healthy fats (eggs, fish, shellfish, nuts, seeds, avocado), g. Fried foods (french fries, fried chicken), h. Foods high in sugar (cookies, candy, ice cream), j. Processed foods (chips, cereals, packaged meals, fast food), i. Foods high in added & saturated fats (butter, oils, pastries, bacon, burgers), j. Water, k. Juice, l. Soda, m. Coffee, n. Tea, o. Milk, p. Energy drink, q. sugarless gum, r. gum with sugar, and s. other. We coded options “a” through “f” for the foods, water, milk, and tea for the beverages, and sugarless gum as healthy. We coded the rest of the categories as unhealthy. The first author of this paper coded the free-response options in the “other” category according to how they best fit into the categories described above. Specific information on how each free response was coded can be found in the analysis code (See [osf.io/p5jbk](https://osf.io/p5jbk/)).

***Social Health.*** Participants responded to whether anyone else was with them at the moment they completed the questionnaire (Yes = 1, No = 0).^1^

***Physical Health.*** Participants responded to the question, “Were you physically active today?” (Yes = 1, No = 0).^1,25^

**Data Analysis**

**Exploratory Analyses.** For the exploratory analyses, we were first going to examine long-term differences, confirm the robustness of significant findings, and ultimately explore mediation and test differences in change from pre-treatment to LTFU. The pre-to-LTFU analyses were exploratory because many of the measures used in the original RCT did not overlap with LTFU. Participants were youths when assessed at baseline and were at least 18 years old at LTFU. Therefore, they could no longer accurately use the same measures as adults that were validated for their age range at baseline. Nevertheless, because of the findings (see Table 2) that TranS-C and PE did not differ significantly at LTFU, we deviated from the pre-registration by not examining mediation or TranS-C vs. PE differences in pre-treatment to LTFU changes in outcomes. Still, we conducted pre-registered moderation analyses, where we added an interaction term of assigned treatment with either age, sex, or family income (see Table S1).

**Calculation of Effect Sizes.** In the multilevel models, we calculated standardized coefficients by standardizing all continuous variables so the regression coefficients can be interpreted as effect sizes in standard deviation units.^37^ In the models using multiple linear regression with continuous independent variables of interest, we calculated standardized coefficients by using the *effectsize* package in R.^38^ For our analyses using multiple linear regression with dichotomous independent variables of interest (i.e., TranS-C vs. PE), we calculated Cohen’s *d* and 95% confidence intervals with the *MOTE* package in R, using *t* values for the effect of TranS-C, relative to PE, obtained from the analyses.^39^

| ***Table S1. Regression Analysis Results on Moderation Effects of Age, Sex Assigned at Birth, and Family Income on TranS-C vs. PE Outcomes*** | | | |
| --- | --- | --- | --- |
| **Age** |  |  |  |
| **Primary Outcomes** | ***d*** | **95% CI** | ***p*** |
| CSM | 0.26 | [-0.13, 0.64] | .193 |
| PROMIS-SRI | -0.15 | [-0.53, 0.23] | .431 |
| PROMIS-SD | 0.09 | [-0.30, 0.47] | .661 |
| Emotional Health Risk | -0.28 | [-0.66, 0.11] | .161 |
| Cognitive Health Risk | **-0.48** | **[-0.86, -0.09]** | **.016** |
| Behavioral Health Risk | -0.10 | [-0.48, 0.28] | .598 |
| Social Health Risk | -0.19 | [-0.57, 0.19] | .326 |
| Physical Health Risk | 0.13 | [-0.25, 0.51] | .494 |
| Utilization of Sleep-health behaviors | 0.30 | [-0.09, 0.68] | .132 |
| Habit Formation | 0.26 | [-0.13, 0.64] | .187 |
| **Secondary Outcomes** | ***d*** | **95% CI** | ***p*** |
| PSQI | -0.03 | [-0.41, 0.35] | .862 |
| Midpoint Fluctuation | -0.12 | [-0.50, 0.26] | .531 |
| Sleep Health Composite | -0.15 | [-0.53, 0.24] | .459 |
| ***EMA Outcomes*** | **β** | **95% CI** | ***p*** |
| Emotional Health Risk | **-0.46** | **[-0.86, -0.07]** | **.022** |
| Cognitive Health Risk | -0.01 | [-0.42, 0.41] | .979 |
| Behavioral Health Risk | -0.05 | [-0.47, 0.37] | .823 |
|  | **OR** | **95% CI** | ***p*** |
| Social Health Risk | 0.51 | [0.22, 1.19] | .121 |
| Physical Health Risk | 0.92 | [0.38, 2.21] | .854 |
| **Other Outcomes** | ***d*** | **95% CI** | ***p*** |
| BMI | -0.31 | [-0.69, 0.07] | .112 |
| Waist Circumference | -0.23 | [-0.60, 0.16] | .254 |
| **Sex Assigned at Birth** |  |  |  |
| **Primary Outcomes** | ***d*** | **95% CI** | ***p*** |
| CSM | -0.17 | [-0.72, 0.39] | .565 |
| PROMIS-SRI | -0.34 | [-0.89, 0.23] | .242 |
| PROMIS-SD | -0.26 | [-0.81, 0.30] | .373 |
| Emotional Health Risk | -0.11 | [-0.66, 0.45] | .708 |
| Cognitive Health Risk | -0.30 | [-0.85, 0.27] | .305 |
| Behavioral Health Risk | -0.25 | [-0.80, 0.31] | .390 |
| Social Health Risk | 0.00 | [-0.56, 0.55] | .992 |
| Physical Health Risk | -0.36 | [-0.91, 0.20] | .212 |
| Utilization of Sleep-health behaviors | -0.54 | [-1.09, 0.03] | .065 |
| Habit Formation | -0.34 | [-0.89, 0.23] | .246 |
| **Secondary Outcomes** | ***d*** | **95% CI** | ***p*** |
| PSQI | -0.38 | [-0.93, 0.19] | .193 |
| Midpoint Fluctuation | -0.10 | [-0.65, 0.46] | .733 |
| Sleep Health Composite | 0.30 | [-0.26, 0.85] | .296 |
| ***EMA Outcomes*** | **β** | **95% CI** | ***p*** |
| Emotional Health Risk | 0.31 | [-0.48, 1.09] | .445 |
| Cognitive Health Risk | -0.32 | [-1.14, 0.49] | .436 |
| Behavioral Health Risk | -0.62 | [-1.43, 0.20] | .137 |
|  | **OR** | **95% CI** | ***p*** |
| Social Health Risk | 1.29 | [0.26, 6.48] | .755 |
| Physical Health Risk | 0.25 | [0.05, 1.35] | .108 |
| **Other Outcomes** | ***d*** | **95% CI** | ***p*** |
| BMI | 0.14 | [-0.42, 0.69] | .637 |
| Waist Circumference | 0.29 | [-0.27, 0.84] | .319 |
| **Family Income** |  |  |  |
| **Primary Outcomes** | ***d*** | **95% CI** | ***p*** |
| CSM | -0.05 | [-0.43, 0.33] | .800 |
| PROMIS-SRI | 0.08 | [-0.30, 0.46] | .693 |
| PROMIS-SD | -0.03 | [-0.41, 0.35] | .884 |
| Emotional Health Risk | -0.14 | [-0.52, 0.24] | .472 |
| Cognitive Health Risk | 0.07 | [-0.31, 0.45] | .714 |
| Behavioral Health Risk | -0.08 | [-0.46, 0.30] | .668 |
| Social Health Risk | 0.14 | [-0.25, 0.52] | .492 |
| Physical Health Risk | -0.20 | [-0.58, 0.19] | .315 |
| Utilization of Sleep-health behaviors | 0.11 | [-0.27, 0.49] | .581 |
| Habit Formation | 0.17 | [-0.22, 0.55] | .395 |
| **Secondary Outcomes** | ***d*** | **95% CI** | ***p*** |
| PSQI | -0.06 | [-0.44, 0.33] | .777 |
| Midpoint Fluctuation | -0.37 | [-0.75, 0.02] | .060 |
| Sleep Health Composite | -0.05 | [-0.43, 0.33] | .806 |
| ***EMA Outcomes*** | **β** | **95% CI** | ***p*** |
| Emotional Health Risk | 0.00 | [-0.40, 0.39] | .981 |
| Cognitive Health Risk | 0.28 | [-0.13, 0.69] | .180 |
| Behavioral Health Risk | -0.16 | [-0.57, 0.25] | .446 |
|  | **OR** | **95% CI** | ***p*** |
| Social Health Risk | 2.31 | [0.92, 5.84] | .076 |
| Physical Health Risk | 1.03 | [0.44, 2.43] | .944 |
| **Other Outcomes** | ***d*** | **95% CI** | ***p*** |
| BMI | 0.17 | [-0.21, 0.55] | .384 |
| Waist Circumference | 0.04 | [-0.34, 0.42] | .843 |
| ***Note:*** We covaried treatment (TranS-C vs. PE), participants’ sex assigned at birth, age at LTFU, and text messaging intervention. CSM = Composite Scale of Morningness; BMI = Body Mass Index; EMA = Ecological Momentary Assessment; LTFU = Long-Term Follow-up; PE = Psychoeducation; PHQ-15 = Physical Health Questionnaire-15; PROMIS-SD = Patient-Reported Outcomes Measurement Information System-Sleep Disturbance; PROMIS-SRI = Patient-Reported Outcomes Measurement Information System-Sleep Related Impairment; PSQI = Pittsburgh Sleep Quality Index; TranS-C = Transdiagnostic Sleep and Circadian Intervention. We added an interaction term of assigned treatment (TranS-C or PE) with either age, sex, or family income. Bolded numbers denote significant (p<0.05) results. | | | |

***Table S2.*** ***Inclusion Criteria Operationalizing ‘At Risk’ for the Five Health Domains.***

| Risk Domain | Criteria for Inclusion |
| --- | --- |
| Emotional | ≥ 4 on any of the following items on the CDRS: Difficulty Having Fun, Social Withdrawal, Irritability, Depressed Feelings, Excessive Weeping, *or* a T-score of 61 or above on the MASC-10, based on age group (10-11 years, 12-15 year, 16-19 years) using the MASC-10 Profile. |
| Behavioral | A SSS score greater than 3.93 for males aged 10-13, greater than 3.19 for females aged 10-13, greater than 4.07 for males aged 14-18, or greater than 3.19 for females aged 14-18 *or* taking ADHD medication or the KSADS indicating a diagnosis of ADHD *or* current alcohol or substance abuse assessed with the KSADS. |
| Social | A parent rating their child as "worse" than others the participants age on one or more of the social behavior items (Section VI) from the CBCL. |
| Cognitive | A parent rating their child as “failing” in one or more academic class from CBCL Section VII. |
| Physical | A score of 4 or above on the PHQ-15, six or more days of school absences in the past semester, or a BMI above the 85th percentile for the participant's sex and age. |

*Note.* ADHD = attention-deficit/hyperactivity disorder; BMI = body mass index (the cutoff corresponds to 1 SD above the mean); CBCL = Child Behavior Checklist,^40^ which asks the parent if their child does “worse than,” “average,” or “better than” other teens their age or if the teen is “failing,” “below average,” “average,” or “above average”; CDRS = Child Depression Rating Scale^41^ (the cutoff is commensurate with “clinical symptoms”)^42^; K-SADS = Schedule for Affective Disorders and Schizophrenia for School-Age Children^43^; MASC-10 = Multidimensional Anxiety Scale for Children (the cutoff T score was selected to capture the “slightly elevated” through to the “very elevated” range)^44^; PHQ-15 = Physical Health Ques- tionnaire-15 (the cutoff corresponds to “minimal somatic symptom severity” through to the “high somatic symptom severity” range)^24^; SSS = Sensation Seeking Scale^45^ (the cutoff correspond to at or above 1 SD over the normative average).^15^

| ***Table S3. Absolute Effect Sizes of Multilevel Logistic Regression Analyses of Binary Outcomes*** | | | |
| --- | --- | --- | --- |
| ***TranS-C vs. PE EMA Outcomes*** | **OR** | **95% CI** | ***p*** |
| Social Health Risk | 0.23 | [-0.57, 1.04] | .566 |
| Physical Health Risk | -0.13 | [-0.95, 0.69] | .754 |
| ***EMA Associations with Social Health Risk at LTFU*** | **OR** | **95% CI** | ***p*** |
| CSM | -0.03 | [-0.45, 0.38] | .879 |
| PROMIS-SRI | 0.13 | [-0.29, 0.54] | .548 |
| PROMIS-SD | 0.30 | [-0.14, 0.74] | .183 |
| PSQI | 0.08 | [-0.33, 0.49] | .691 |
| Midpoint Fluctuation | 0.20 | [-0.24, 0.64] | .367 |
| Sleep Health Composite | 0.03 | [-0.38, 0.43] | .903 |
| ***EMA Associations with Physical Health Risk at LTFU*** | **OR** | **95% CI** | ***p*** |
| CSM | -0.18 | [-0.59, 0.23] | .378 |
| PROMIS-SRI | 0.04 | [-0.36, 0.44] | .852 |
| PROMIS-SD | -0.22 | [-0.65, 0.21] | .313 |
| PSQI | 0.08 | [-0.33, 0.49] | .699 |
| Midpoint Fluctuation | -0.22 | [-0.65, 0.21] | .321 |
| Sleep Health Composite | 0.06 | [-0.34, 0.46] | .776 |
| ***Moderation Effects of TranS-C vs. PE EMA Outcomes*** | |  |  |
| *Age* | **OR** | **95% CI** | ***p*** |
| Social Health Risk | 0.51 | [0.22, 1.19] | .121 |
| Physical Health Risk | -0.08 | [-0.96, 0.79] | .854 |
| *Sex Assigned at Birth* | **OR** | **95% CI** | ***p*** |
| Social Health Risk | 0.26 | [-1.36, 1.87] | .755 |
| Physical Health Risk | -1.38 | [-3.05, 0.30] | .108 |
| *Family Income* | **OR** | **95% CI** | ***p*** |
| Social Health Risk | 0.84 | [-0.09, 1.76] | .076 |
| Physical Health Risk | 0.03 | [-0.83, 0.89] | .944 |

*Note.* We covaried sex assigned at birth, age at LTFU, and text messaging intervention in all analyses. In EMA associations with social and physical health risks, we also covaried for assigned treatment (TranS-C or PE). For moderation effects, we added an interaction term of assigned treatment (TranS-C or PE) with either age, sex, or family income. CSM = Composite Scale of Morningness; BMI = Body Mass Index; EMA = Ecological Momentary Assessment; LTFU = Long-Term Follow-up; PE = Psychoeducation; PHQ-15 = Physical Health Questionnaire-15; PROMIS-SD = Patient-Reported Outcomes Measurement Information System-Sleep Disturbance; PROMIS-SRI = Patient-Reported Outcomes Measurement Information System-Sleep Related Impairment; PSQI = Pittsburgh Sleep Quality Index; TranS-C = Transdiagnostic Sleep and Circadian Intervention.

| **Table S4. Descriptive Statistics** | | | | |
| --- | --- | --- | --- | --- |
|  | **TranS-C** | | **PE** | |
| **Primary Outcomes** | ***M*** | ***SD*** | ***M*** | ***SD*** |
| CSM | 30.65 | 8.58 | 31.13 | 5.92 |
| PROMIS-SRI | 19.88 | 6.34 | 20.49 | 6.08 |
| PROMIS-SD | 19.12 | 5.29 | 20.20 | 5.28 |
| ***Self-Report Composite Risk Score*** | ***M*** | ***SD*** | ***M*** | ***SD*** |
| *Emotional Health Risk* |  |  |  |  |
| DASS | 16.16 | 10.96 | 14.95 | 9.29 |
| Composite | 0.06 | 1.09 | -0.06 | 0.92 |
| *Cognitive Health Risk* |  |  |  |  |
| PROMIS-CF | 44.78 | 7.41 | 45.08 | 7.75 |
| PROMIS-CF-A | 48.89 | 7.36 | 46.91 | 7.15 |
| Composite | -46.83 | 6.59 | -45.99 | 6.89 |
| *Behavioral Health Risk* |  |  |  |  |
| BSSS | 25.37 | 6.17 | 26.27 | 5.90 |
| PROMIS-AU | 45.60 | 8.45 | 46.24 | 7.40 |
| PROMIS-SSU | 44.64 | 5.06 | 44.05 | 4.53 |
| Composite | 57.80 | 7.18 | 58.28 | 6.08 |
| *Social Health Risk* |  |  |  |  |
| PROMIS-C | 53.51 | 7.45 | 52.19 | 7.78 |
| PROMIS-ES | 55.10 | 8.11 | 54.65 | 6.66 |
| PROMIS-SI | 50.47 | 7.48 | 50.24 | 7.39 |
| PROMIS-AP | 48.76 | 6.05 | 49.40 | 6.28 |
| Composite | -26.72 | 5.79 | -26.50 | 5.26 |
| *Physical Health Risk* |  |  |  |  |
| MAQ | 20.31 | 23.63 | 29.37 | 46.98 |
| PHQ-15 | 8.48 | 4.07 | 7.98 | 4.25 |
| Composite | 0.08 | 0.65 | -0.05 | 0.78 |
| Utilization of Sleep-health behaviors | 29.55 | 8.12 | 27.93 | 6.58 |
| Habit Formation | 26.82 | 8.92 | 24.61 | 7.68 |
| **Secondary Outcomes** | ***M*** | ***SD*** | ***M*** | ***SD*** |
| PSQI | 7.39 | 2.48 | 7.47 | 3.14 |
| Midpoint Fluctuation | 0.81 | 0.41 | 0.8 | 0.4 |
| Sleep Health Composite | 3.84 | 1.39 | 3.89 | 1.31 |
| **Other Outcomes** | ***M*** | ***SD*** | ***M*** | ***SD*** |
| BMI | 24.4 | 5.52 | 25.52 | 5.37 |
| Waist Circumference (cm) | 83.28 | 16.12 | 86.59 | 17.32 |
| ***EMA Composite Risk Score*** | ***M*** | ***SD*** | ***M*** | ***SD*** |
| Emotional Health Risk | 5.04 | 2.36 | 5.60 | 2.33 |
| Cognitive Health Risk | 6.33 | 2.22 | 6.62 | 1.97 |
| Behavioral Health Risk | 3.80 | 5.27 | 4.13 | 5.96 |
| Social Health Risk | 0.47 | 0.50 | 0.49 | 0.50 |
| Physical Health Risk | 0.51 | 0.51 | 0.46 | 0.50 |
| ***Note:***  BMI = Body Mass Index BSSS = Brief Sensation Seeking Scale; CSM = Composite Scale of Morningness; DASS = Depression, Anxiety and Stress Scales; MAQ = Modifiable Activity Questionnaire; PE = Psychoeducation; PHQ-15 = Physical Health Questionnaire-15; PROMIS-AP = Patient-Reported Outcomes Measurement Information System-Ability to Participate; PROMIS-AU = Patient-Reported Outcomes Measurement Information System-Alcohol Use; PROMIS-C = Patient-Reported Outcomes Measurement Information System-Companionship; PROMIS-CF = Patient-Reported Outcomes Measurement Information System-Cognitive Function; PROMIS-CF-A = Patient-Reported Outcomes Measurement Information System-Cognitive Function – Abilities; PROMIS-ES = Patient-Reported Outcomes Measurement Information System-Emotional Support; PROMIS-SI = Patient-Reported Outcomes Measurement Information System-Social Isolation; PROMIS-SD = Patient-Reported Outcomes Measurement Information System-Sleep Disturbance; PROMIS-SRI = Patient-Reported Outcomes Measurement Information System-Sleep Related Impairment; PROMIS-SSU = Patient-Reported Outcomes Measurement Information System-Severity of Substance Use; PSQI = Pittsburgh Sleep Quality Index; TranS-C = Transdiagnostic Sleep and Circadian Intervention. | | | | |

**Supplemental References**

1. Harvey AG, Hein K, Dolsen MR, et al. Modifying the impact of eveningness chronotype (“Night-Owls”) in youth: A randomized controlled trial. *Journal of the American Academy of Child & Adolescent Psychiatry*. 2018;57(10):742-754.

2. Maslowsky J, Ozer EJ. Developmental trends in sleep duration in adolescence and young adulthood: evidence from a national United States sample. *Journal of Adolescent Health*. 2014;54(6):691-697.

3. Giannotti F, Cortesi F. Sleep patterns and daytime function in adolescence: An epidemiological survey of an Italian high school student sample. In: *Adolescent Sleep Patterns: Biological, Social, and Psychological Influences*. Cambridge University Press; 2002:132-147. doi:10.1017/CBO9780511499999.011

4. Dong L, Martinez AJ, Buysse DJ, Harvey AG. A Composite Measure of Sleep Health Predicts Concurrent Mental and Physical Health Outcomes in Adolescents Prone to Eveningness. *Sleep Health*. 2019;5(2):166-174. doi:10.1016/j.sleh.2018.11.009

5. Chaput JP, Dutil C, Sampasa-Kanyinga H. Sleeping hours: what is the ideal number and how does age impact this? *Nat Sci Sleep*. 2018;10:421-430. doi:10.2147/NSS.S163071

6. Griggs S, Pignatiello G, Hickman Jr RL. A composite measure of sleep health is associated with glycaemic target achievement in young adults with type 1 diabetes. *Journal of Sleep Research*. 2023;32(3):e13784. doi:10.1111/jsr.13784

7. Lee S, Mu CX, Wallace ML, et al. Sleep health composites are associated with the risk of heart disease across sex and race. *Sci Rep*. 2022;12(1):2023. doi:10.1038/s41598-022-05203-0

8. Carney CE. The consensus sleep diary: Standardizing prospective sleep self-monitoring. *Sleep*. 2012;35:287-302.

9. Coates TJ, Killen JD, George J, Marchini E, Silverman S. Estimating sleep parameters: a multitrait-multimethod analysis. *Journal of Consulting and Clinical Psychology*. 1982;50:345-352.

10. Exelmans L, Van den Bulck J. Bedtime, shuteye time and electronic media: sleep displacement is a two‐step process. *Journal of Sleep Research*. 2017;26(3):364-370.

11. Lovibond PF, Lovibond SH. The structure of negative emotional states: comparison of the Depression Anxiety Stress Scales (DASS) with the Beck Depression and Anxiety Inventories. *Behav Res Ther*. 1995;33(3):335-343. doi:10.1016/0005-7967(94)00075-u

12. Iverson GL, Marsh JM, Connors EJ, Terry DP. Normative Reference Values, Reliability, and Item-Level Symptom Endorsement for the PROMIS® v2.0 Cognitive Function-Short Forms 4a, 6a and 8a. *Archives of Clinical Neuropsychology*. 2021;36(7):1341-1349. doi:10.1093/arclin/acaa128

13. Becker H, Stuifbergen A, Lee H, Kullberg V. Reliability and Validity of PROMIS Cognitive Abilities and Cognitive Concerns Scales Among People with Multiple Sclerosis. *International Journal of MS Care*. 2014;16(1):1-8. doi:10.7224/1537-2073.2012-047

14. Meredith SE, Sweeney MM, Johnson PS, Johnson MW, Griffiths RR. Weekly energy drink use is positively associated with delay discounting and risk behavior in a nationwide sample of young adults. *Journal of Caffeine Research*. 2016;6(1):10-19.

15. Stephenson MT, Hoyle RH, Palmgreen P, Slater MD. Brief measures of sensation seeking for screening and large-scale surveys. *Drug and Alcohol Dependence*. 2003;72(3):279-286.

16. Pilkonis PA, Yu L, Dodds NE, Johnston KL, Lawrence SM, Daley DC. Validation of the alcohol use item banks from the Patient-Reported Outcomes Measurement Information System (PROMIS®). *Drug and Alcohol Dependence*. 2016;161:316-322. doi:10.1016/j.drugalcdep.2016.02.014

17. Pilkonis PA, Yu L, Dodds NE, et al. Item banks for substance use from the Patient-Reported Outcomes Measurement Information System (PROMIS®): Severity of use and positive appeal of use. *Drug and Alcohol Dependence*. 2015;156:184-192. doi:10.1016/j.drugalcdep.2015.09.008

18. Hahn EA, DeWalt DA, Bode RK, et al. New English and Spanish Social Health Measures Will Facilitate Evaluating Health Determinants. *Health Psychol*. 2014;33(5):490-499. doi:10.1037/hea0000055

19. Nieforth LO, Miller EA, MacDermid Wadsworth S, O’Haire ME. Posttraumatic stress disorder service dogs and the wellbeing of veteran families. *European Journal of Psychotraumatology*. 2022;13(1):2062997. doi:10.1080/20008198.2022.2062997

20. Cella D, Riley W, Stone A, et al. The Patient-Reported Outcomes Measurement Information System (PROMIS) developed and tested its first wave of adult self-reported health outcome item banks: 2005–2008. *Journal of Clinical Epidemiology*. 2010;63(11):1179-1194. doi:10.1016/j.jclinepi.2010.04.011

21. Aaron DJ, Kriska AM. Modifiable activity questionnaire for adolescents. *Medicine and Science in Sports and Exercise*. 1997;29:s79-s82.

22. Interian A, Allen LA, Gara MA, Escobar JI, Díaz-Martínez AM. Somatic complaints in primary care: further examining the validity of the Patient Health Questionnaire (PHQ-15). *Psychosomatics*. 2006;47(5):392-398.

23. Kocalevent RD, Hinz A, Brähler E. Standardization of a screening instrument (PHQ-15) for somatization syndromes in the general population. *BMC Psychiatry*. 2013;13(1):91.

24. Kroenke K, Spitzer RL, Williams JB. The PHQ-15: validity of a new measure for evaluating the severity of somatic symptoms. *Psychosomatic medicine*. 2002;64(2):258-266.

25. Silk JS, Forbes EE, Whalen DJ, et al. Daily emotional dynamics in depressed youth: A cell phone ecological momentary assessment study. *Journal of Experimental Child Psychology*. 2011;110(2):241-257.

26. Laurent J, Catanzaro SJ, Joiner TE, et al. A measure of positive and negative affect for children: Scale development and preliminary validation. *Psychological Assessment*. 1999;11(3):326-338.

27. Diener E. Subjective well-being: The science of happiness and a proposal for a national index. *American Psychologist*. 2000;55:34-43. doi:10.1037/0003-066X.55.1.34

28. Fredrickson BL, Losada MF. Positive affect and the complex dynamics of human flourishing. *American Psychologist*. 2005;60:678-686. doi:10.1080/17439760500510981

29. Martin F, Oliver T. Behavioral activation for children and adolescents: a systematic review of progress and promise. *Eur Child Adolesc Psychiatry*. 2019;28(4):427-441. doi:10.1007/s00787-018-1126-z

30. Whalen DJ, Silk JS, Semel M, et al. Caffeine consumption, sleep, and affect in the natural environments of depressed youth and healthy controls. *Journal of Pediatric Psychology Special Issue: Sleep in Pediatric Medical Populations*. 2008;33:358-357. doi:doi:10.1093/jpepsy/jsm086

31. Miller T, Hendrie D. *Substance Abuse Prevention Dollars and Cents: A Cost-Benefit Analysis*. Substance Abuse and Mental Health Services Administration; 2008.

32. Trogdon JG, Finkelstein EA, Hylands T, Dellea PS, Kamal-Bahl SJ. Indirect costs of obesity: a review of the current literature. *Obes Rev*. 2008;9(5):489-500. doi:10.1111/j.1467-789X.2008.00472.x

33. Ward ZJ, Bleich SN, Long MW, Gortmaker SL. Association of body mass index with health care expenditures in the United States by age and sex. *PLOS ONE*. 2021;16(3):e0247307. doi:10.1371/journal.pone.0247307

34. CPI Inflation Calculator. Published 2023. Accessed February 24, 2023. https://www.bls.gov/data/inflation_calculator.htm

35. Nutt DJ, King LA, Phillips LD. Drug harms in the UK: a multicriteria decision analysis. *The Lancet*. 2010;376(9752):1558-1565. doi:10.1016/S0140-6736(10)61462-6

36. van Amsterdam J, Opperhuizen A, Koeter M, van den Brink W. Ranking the harm of alcohol, tobacco and illicit drugs for the individual and the population. *Eur Addict Res*. 2010;16(4):202-207. doi:10.1159/000317249

37. Lorah J. Effect size measures for multilevel models: definition, interpretation, and TIMSS example. *Large-scale Assess Educ*. 2018;6(1):8. doi:10.1186/s40536-018-0061-2

38. Ben-Shachar M, Lüdecke D, Makowski D. effectsize: Estimation of Effect Size Indices and Standardized Parameters. *JOSS*. 2020;5(56):2815. doi:10.21105/joss.02815

39. Buchanan EM, Gillenwaters AM, Scofield JE, Valentine KD. MOTE: Effect Size and Confidence Interval Calculator. Published online April 10, 2019. Accessed June 27, 2023. https://cran.r-project.org/web/packages/MOTE/index.html

40. Becker SP, Ramsey RR, Byars KC. Convergent validity of the Child Behavior Checklist sleep items with validated sleep measures and sleep disorder diagnoses in children and adolescents referred to a sleep disorders center. *Sleep Med*. 2015;16(1):79-86. doi:10.1016/j.sleep.2014.09.008

41. Poznanski EO, Grossman JA, Buchsbaum Y, Banegas M, Freeman L, Gibbons R. Preliminary studies of the reliability and validity of the children’s depression rating scale. *J Am Acad Child Psychiatry*. 1984;23(2):191-197. doi:10.1097/00004583-198403000-00011

42. Poznanski E, Freeman L, Mokros H. Childrens Depression Rating Scale–Revised. *Psychopharmacol Bull*. 1985;21:979-989.

43. Kaufman J, Birmaher B, Brent D, et al. Schedule for Affective Disorders and Schizophrenia for School-Age Children-Present and Lifetime Version (K-SADS-PL): initial reliability and validity data. *J Am Acad Child Adolesc Psychiatry*. 1997;36(7):980-988. doi:10.1097/00004583-199707000-00021

44. March JS, Sullivan K. Test-retest reliability of the Multidimensional Anxiety Scale for Children. *J Anxiety Disord*. 1999;13(4):349-358. doi:10.1016/s0887-6185(99)00009-2

45. Russo MF, Stokes GS, Lahey BB, et al. A sensation seeking scale for children: Further refinement and psychometric development. *J Psychopathol Behav Assess*. 1993;15(2):69-86. doi:10.1007/BF00960609
